# Altered Iron Metabolism and Impact in Cancer Biology, Metastasis, and Immunology

**DOI:** 10.3389/fonc.2020.00476

**Published:** 2020-04-09

**Authors:** Rikki A. M. Brown, Kirsty L. Richardson, Tasnuva D. Kabir, Debbie Trinder, Ruth Ganss, Peter J. Leedman

**Affiliations:** ^1^Queen Elizabeth II Medical Centre, Harry Perkins Institute of Medical Research, Perth, WA, Australia; ^2^UWA Centre for Medical Research, University of Western Australia, Perth, WA, Australia; ^3^UWA Medical School, University of Western Australia, Perth, WA, Australia

**Keywords:** iron metabolism, cancer biology, metastasis, microRNAs, iron chelator, ferroptosis, tumor microenvironment, drug resistance

## Abstract

Iron is an essential nutrient that plays a complex role in cancer biology. Iron metabolism must be tightly controlled within cells. Whilst fundamental to many cellular processes and required for cell survival, excess labile iron is toxic to cells. Increased iron metabolism is associated with malignant transformation, cancer progression, drug resistance and immune evasion. Depleting intracellular iron stores, either with the use of iron chelating agents or mimicking endogenous regulation mechanisms, such as microRNAs, present attractive therapeutic opportunities, some of which are currently under clinical investigation. Alternatively, iron overload can result in a form of regulated cell death, ferroptosis, which can be activated in cancer cells presenting an alternative anti-cancer strategy. This review focuses on alterations in iron metabolism that enable cancer cells to meet metabolic demands required during different stages of tumorigenesis in relation to metastasis and immune response. The strength of current evidence is considered, gaps in knowledge are highlighted and controversies relating to the role of iron and therapeutic targeting potential are discussed. The key question we address within this review is whether iron modulation represents a useful approach for treating metastatic disease and whether it could be employed in combination with existing targeted drugs and immune-based therapies to enhance their efficacy.

## Introduction

Iron is an essential element utilized by living cells during many cellular processes. However, evidence links iron to various diseases including cancer. The biological activity of iron stems from cycling between ferrous (Fe^2+^) and ferric (Fe^3+^) states by accepting or donating electrons in cellular reactions. Efficient electron transfer underlies its importance as an enzyme cofactor, many of which are involved in DNA replication. Iron bioavailability is, therefore, rate-limiting during DNA synthesis and cells which undergo rapid division require more iron. It is, therefore, not surprising that iron accumulation is often observed in tumor tissues. Recently, iron accumulation at sites of chronic inflammation was proposed as a root cause of malignancy (1). Excess Fe^2+^ and H_2_O_2_ participate in Fenton reactions, generating reactive oxygen species (ROS), ·OH and OH–. Glycolytic ATP generation and nucleotide synthesis are increased to neutralize excess OH^−^, which drives DNA synthesis and cell division (1). Furthermore, hydroxyl radicals can cause changes that lead to persistent inflammation and cell survival/proliferation signals (1). Yet, hydroxyl radicals can also damage lipids in the cell membrane triggering ferroptosis (2). This iron-dependent form of cell death represents a potential strategy to inhibit tumor growth. Therefore, while iron accumulation may be conducive to malignant transformation or iron-dependent cell death, maintaining stable iron levels is necessary for cancer progression.

Metastasis is the major contributor to cancer mortality and morbidity. Over 90% of cancer-related deaths are due to metastases (3). Metastatic disease is rarely treated effectively with surgery alone, so patients receive systemic treatments, such as chemotherapies, targeted and immune-based therapies. However, drug resistance is common and, hence, many cancers will continue to progress or recur. Iron plays a role in initiating and supporting metastasis in several ways. While a single genetic mutation, amplification or deletion is insufficient to cause metastasis, the accumulation of ROS through Fenton reactions can stimulate widespread modifications to DNA, proteins and lipids which promotes a more aggressive tumor phenotype. ROS induce metabolic rewiring in cancer cells toward glycolysis, a feature described as the “Warburg effect,” however, the byproducts of this process increase intracellular acidity and in response, protons are exported into the extracellular space creating an acidic microenvironment (4). The acidic environment breaks down the extracellular matrix (ECM), promotes neo-vascularization, suppresses T cell activity and induces migration and invasion (4). Innate immune cells and cancer associated fibroblasts are also a major source of iron and ROS, essentially adding fuel to the fire and creating the perfect storm for a reaction that cannot be biologically regulated. This review will explain cellular iron metabolism and homeostasis mechanisms that go awry to support tumor growth and progression as well as potential iron-based therapeutic strategies to treat cancer.

## Cellular Iron Metabolism

**Figure 1:**
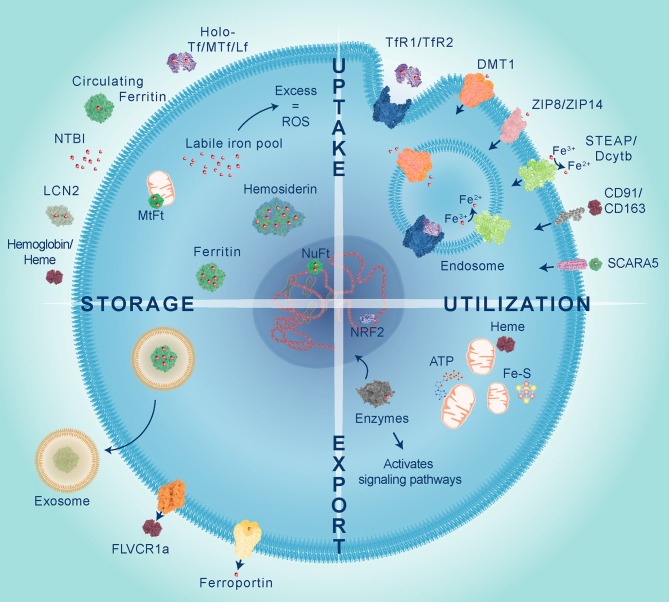
Iron is transported to cells bound to proteins belonging to the transferrin family including transferrin (Tf), melanotransferrin (MTf), and lactoferrin (Lf), circulating ferritin, lipocalin 2 (LCN2) or integrated in heme proteins, such as hemoglobin. Iron uptake predominantly occurs through endocytosis of transferrin receptors (TfR1 or TfR2) bound to Tf. Within the endosome iron is released from Tf, reduced by STEAP metalloreductases and exported into the cytoplasm via divalent metal transporter 1 (DMT1). Non-transferrin bound iron (NTBI), which has been reduced by STEAP or Dcytb, can be directly transported by DMT1, Zrt-, and Irt-like protein 8 and 14 (ZIP8 and ZIP14). Heme iron is taken up by scavenger receptors clusters of differentiation 91 and 163 (CD91 and CD163), whilst circulating ferritin is imported via scavenger receptor class A member 5 (SCARA5). Iron is utilized within the mitochondria for heme and Fe-S cluster synthesis which are important for ATP production. Iron is used as a co-factor for numerous enzymes to activate cell signaling and control gene expression, such as NRF2 which activates transcription of antioxidant genes in response to oxidative stress. Excess labile iron gives rise to reactive oxygen species (ROS), therefore iron is safely stored within different cellular compartments by cytoplasmic ferritin, nuclear ferritin (NuFt), and mitochondrial ferritin (MtFt) or in some cases forms aggregates termed hemosiderin. Intracellular iron levels can be reduced by efflux via ferroportin, export of heme by feline leukemia virus subgroup C cellular receptor 1a (FLVCR1a) or released in exosomes bound to ferritin.

Iron metabolism involves tightly controlled cellular uptake, utilization, storage and export mechanisms, as illustrated in Figure 1. Most iron is stored in red blood cells and is a major source of systemic iron through their degradation, releasing iron from heme and making it available for other cells to utilize (5). Dietary iron uptake occurs through divalent metal ion transporter 1 (DMT1) expressed on enterocytes in the duodenum and upper ilium in the small intestine (6). Iron is transported from the sites of absorption to other tissues predominantly by binding to the protein transferrin (Tf). Tf binds to transferrin receptors, TfR1 or TfR2, and the complex is internalized by endocytosis. Inside the endosome, the acidic environment (pH 5.5) facilitates dissociation of iron from the complex. Iron is reduced by six-transmembrane epithelial antigen of the prostate 3 (STEAP3) and exported by DMT1 into the intracellular labile iron pool (LIP) to be utilized, stored, or oxidized by ceruloplasmin and exported from the cell by ferroportin. TfR is either recycled to internalize more Tf-Fe or degraded. Iron is also bound to other ligands termed non-transferrin bound iron (NTBI), circulating ferritin and hemoglobin/heme, can also transport iron and are taken up by cells using different mechanisms which are described in more detail below.

Iron utilization occurs within the mitochondria, cytoplasm and nucleus. Iron is required in the mitochondria for synthesis of heme and Fe-S clusters, both of which are essential cofactors in energy production through transfer of electrons between mitochondrial respiratory complexes (7). Shifts in redox state of Fe-S clusters also act as a surveillance mechanism to detect DNA damage (8, 9). Outside of its role in the mitochondria, iron acts as an essential cofactor for the activity of many enzymes. For example, deoxyhypusine hydroxylase (DOHH) is a cytoplasmic iron-dependent enzyme that catalyzes the addition of a unique amino acid called hypusine, to eukaryotic initiation factor 5A (eIF5A), thereby coordinating its activity during protein translation and is an important process for controlling cell growth and mRNA decay (10). The nuclear enzyme ribonucleotide reductase (RNR) requires iron to mediate synthesis of deoxyribonucleotides, the building blocks used for DNA replication and repair (9). Given that iron is required in different cell compartments multiple reservoirs exist with iron safely stored and released as required.

Excess intracellular iron is primarily stored in the form of ferritin. It is a 24-mer complex made up of ferritin heavy chain 1 (FTH1) and ferritin light chain (FTL) subunits that form a hollow nanocage, storing ~4,500 Fe^3+^ atoms per complex (11). Although similar in sequence (55% shared) the two ferritin subunits have different functions and their ratios differ in tissues. Organs with high iron turnover (e.g., heart) contain more FTH1 as it possesses ferroxidase activity, while organs that store iron (e.g., liver) have more FTL which facilitates the storage of iron in the core (12). Nuclear ferritin protects DNA by sequestering free iron and releases it to activate iron-dependent enzymes and relax the DNA structure in preparation for synthesis (12). Nuclear and cytoplasmic ferritins are the same, although they do not contain a nuclear localization signal, so the mechanism of translocation remains unclear (12). In contrast, mitochondrial ferritin (MtFt) contains a mitochondrial targeting sequence and lacks an iron-responsive element (IRE) and, thus, it is not subject to the same regulatory mechanisms as other ferritins (13). Iron accumulates in mitochondria as a result of defective heme and Fe-S cluster synthesis, yet it is still unclear whether cytosolic iron levels influence iron accumulation in mitochondria. It is likely that because MtFt lacks iron regulation, levels that exceed MtFt storage capacity will give rise to Fenton reaction-induced ROS leading to diseases including cancer.

## Iron Homeostasis

**Figure 2:**
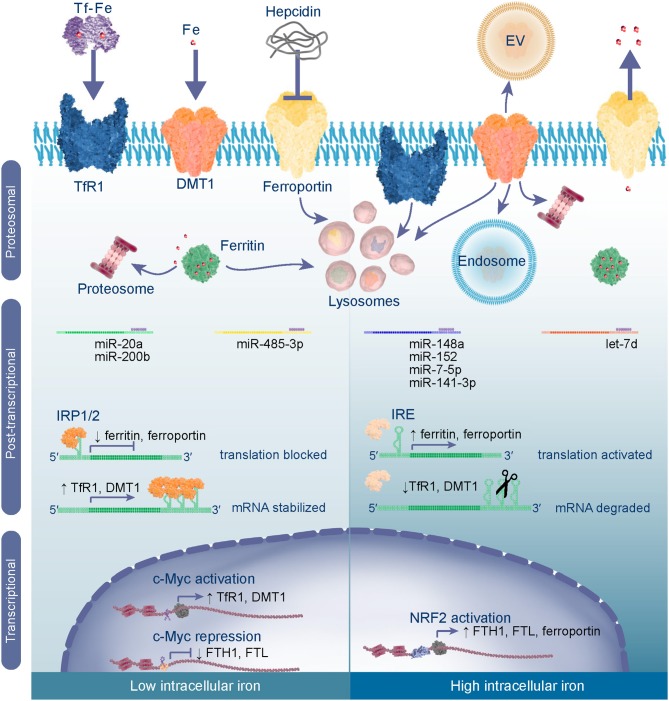
Iron metabolism is regulated through transcriptional, post-transcriptional and proteosomal mechanisms. In low intracellular iron conditions c-Myc acts as a transcriptional activator of iron import genes TfR1 and DMT1 and represses expression of ferritin to increase the intracellular labile iron pool (LIP). In the cytoplasm iron regulatory proteins 1 and 2 (IRP1/IRP2) bind to iron responsive elements (IREs) in the 5′-UTR of ferritin and ferroportin mRNAs blocking their translation, whilst binding to 3′-UTR IREs stabilizes TfR1 and DMT1 mRNAs ensuring their translation. mRNAs may also be subject to post-transcriptional control by specific microRNAs (miRs) which bind to the 3′-UTR to inhibit translation or induce degradation of the transcript. To reduce systemic iron levels hepcidin is released by liver cells and targets ferroportin for lysosomal degradation thereby reducing export of iron into the blood stream. Degradation of ferritin is a mechanism for controlling intracellular labile iron levels by undergoing proteosomal or lysosomal degradation, to liberate iron from the nanocage and reduce apo-protein levels. When intracellular iron is high and oxidative stress is imminent NRF2 activates transcription of ferritin and ferroportin genes. In this case the IRPs are degraded and, hence, their translation is activated, whilst TfR1 and DMT1 mRNAs undergo endonuclease attack or are downregulated by miRs. Excess iron is stored in ferritin or exported from the cell via ferroportin. Further iron import is inhibited by degradation of TfR1 and DMT1 proteins or release from the cell membrane [internalization in the endosome or release in extracellular vesicles (EV)].

Iron homeostasis is achieved through regulating gene transcription, protein synthesis, and degradation (Figure 2). Metabolism of iron and oxygen are inexplicably linked and share some of the same regulatory mechanisms which are reviewed by Renassia et al. (14), Shah et al. (15), and in the context of cancer by Pfeifhofer-Obermair et al. (16). When iron homeostasis is disrupted excess levels cause oxidative stress resulting from an imbalance between the abundance of ROS and antioxidants. ROS are targeted by antioxidant systems which reduce them to non-reactive H_2_O before damage ensues (17). The transcription factor, nuclear factor erythroid 2-related factor 2 (NRF2), is a master regulator of oxidative stress (18). In response to oxidative stress NRF2 translocates into nucleus and activates gene expression. This results in increased expression of antioxidant proteins to reduce ROS, as well as ferritins and ferroportin to reduce the LIP and prevent further ROS formation. Besides being destructive, ROS also act as a signaling molecule activating pathways, such as the epidermal growth factor receptor (EGFR) pathway (19). Downstream of EGFR are PI3K-Akt (Akt) and mitogen-activated protein kinases (MAPK) which activate mammalian target of rapamycin (mTOR) and proto-oncogene c-Myc (c-Myc) that are often hyper-activated in cancers. C-Myc is a transcription factor that represses expression of FTH1/FTL and activates expression of TfR1 and DMT1 to increase the intracellular LIP (20), and therefore provides a link between oncogenic signaling and iron metabolism.

mRNAs that contain IREs are subject to control by iron regulatory proteins, IRP1 and IRP2, which act in response to cellular iron levels. IREs are stem-loop structures that are present within the 5′- or 3′-untranslated region (UTR) of mRNAs (5). In low iron conditions (Figure 2), binding of IRP1 or IRP2 to IREs located in the 5′-UTR inhibits translation of mRNAs, such as ferritin or ferroportin by blocking the recruitment of ribosomes. Alternatively, binding of IRPs to the 3′-UTR stabilizes mRNAs including TfR1 and DMT1. The net effect is an increase in the LIP through reduced synthesis of iron export/storage proteins and an increase of iron importers. In contrast, high labile iron induces proteosomal degradation of IRPs, such that translation of ferritin mRNAs and ferroportin are unobstructed, while mRNAs with 3′-UTR IREs are subject to endonuclease attack and degraded. Therefore, homeostasis is maintained by producing more proteins for iron efflux over influx and ferritin is made available to store iron and prevent oxidative stress. Iron sensing transpires through Fe^2+^ acting directly on the IRPs and by binding to IREs, causing a conformational change that impairs affinity of IRP for IREs (21). This indicates that IRP activity, and IRE structure and location coordinately determine expression of these genes and presents one type of exquisite post-transcriptional control.

Gene expression is also controlled at the post-transcription level by microRNAs (miRNAs). miRNAs are short (~22 nucleotides) non-coding RNAs which control gene expression through targeting mRNAs for degradation or repressing their translation (22). Hence, miRNAs regulate many genes including those involved in iron metabolism (Figure 2), but miRNAs are also controlled by iron levels. For instance, intestinal iron absorption by enterocytes can be regulated by levels of DMT1 which is a target of the miRNA, let-7d (23). The miRNA biogenesis pathway is also subject to regulation, modifying the abundance and function of miRNAs and can be affected by intracellular iron levels (24). To elaborate, poly(C)-binding protein 2 (PCBP2) functions by forming a multimeric complex which binds to miRNA precursors and presents them to DICER for processing into mature sequences, but excess iron impairs PCBP2 activity, thereby reducing the abundance of mature miRNAs. This is relevant because some miRNAs function as tumor suppressors, consequently their loss removes the brake on expression of oncogenes that drive transformation and tumor progression. Li et al. found that iron chelators can enhance processing of miRNA precursors by promoting PCBP2 multimerization and subsequent association of PCBP2 with the precursors and DICER processing (24). Therefore, due to their reciprocal relationship, miRNA mimics could be used to regulate iron metabolism or iron chelation could be used to promote expression and function of tumor suppressor miRNAs.

Iron homeostasis is maintained through protein degradation pathways. Hepcidin is a peptide hormone that controls systemic iron levels by inducing ferroportin degradation (Figure 2). When systemic iron is high, hepcidin is released by the liver into the circulation which induces internalization, ubiquitination, and degradation of ferroportin in lysosomes to prevent the release of iron from cells (25). Conversely, when systemic iron is low, ferroportin isn't targeted for degradation permitting iron export into the blood stream (26). This mechanism is important for duodenal enterocytes to control dietary iron absorption, macrophages which recycle iron from senescent erythrocytes, and hepatocytes which store/release iron as required (26). High ferroportin has also been linked to ferritin degradation (27). Ferritin is degraded through lysosomal or proteosomal mechanisms depending whether degradation is necessary to liberate iron or because ferritin isn't required (28, 29). For example, agents which reduce intracellular iron (e.g., membrane-permeable iron chelators) induce proteasomal degradation of ferritin, whilst those that limit iron uptake (e.g., impermeable iron chelators) promote degradation via the lysosome and activate autophagy (27). Iron import is also controlled by lysosomal or proteasomal degradation of TfR1 and DMT1 or by release from the plasma membrane into extracellular vesicles or endosomes (6, 30, 31). Therefore, post-translational mechanisms are another level of control to ensure iron homeostasis.

## Altered Iron Metabolism in Tumors

Altered iron metabolism is considered a hallmark of cancer (32–34). Increased intercellular iron import and reduced iron export is common in many cancers, but dysregulation can occur at all stages of iron metabolism. Table 1 summarizes altered expression of iron-related proteins in cancers and their potential prognostic value.

**Table 1:** Expression of iron metabolism related proteins and relevance to cancer.

**Protein**	**Sample type**	**Relevance in cancer**
Transferrin (Tf)	Serum	High Tf saturation correlated with increased risk of colorectal, lung, and breast cancers and mortality from these cancers (35, 36).
		Low Tf saturation and high Fe binding capacity correlated with increased risk of stomach cancer (35).
Melanotransferrin (MTf)	Cell lines	High expression in melanoma and breast cancer lines (37).
	Tissue samples	Highly expressed in melanoma tissues, but is also detectable in breast, liposarcoma, and lung cancer tissues (37). High expression correlated with high tumor grade and lymph node metastases of colorectal cancer tissues (38).
	Serum	High levels detected in colorectal cancer patients (38).
Lactoferrin (Lf)	Cell lines	Low in some prostate lines due to hypermethylation of promoter (39).
	Tissues	Low/absence of Lf associated with shorter PFS^*^ of breast and prostate cancers (39, 40). Lf lower in gastric cancer samples compared to normal adjacent tissues (41). Lf lower in nasopharyngeal carcinomas than matched normal samples and expression negatively correlated with disease stage (42).
	Serum	Patients with prostate cancer had significantly lower levels of Lf compared to healthy controls (39).
Lipocalin 2 (LCN2)	Cell lines	High expression observed in ovarian (43), thyroid (44), breast (45), and colorectal (46) cancer cell lines.
	Tissue samples	Highly elevated in ovarian, thyroid, colorectal, and liver cancers compared non-tumor tissues (43, 44, 46, 47). Expression positively correlated with breast and thyroid tumor grade (44, 45).
	Serum	Higher in ovarian and liver cancer patients compared with healthy controls and predictive of poor OS^*^ for ovarian cancer (43, 46).
	Urine	Higher in breast cancer patients than healthy controls (45).
Transferrin receptor 1 (TfR1)	Cell lines	Overexpressed in breast, colon, prostate, leukemia, and esophageal cancer cells (48, 49).
	Tissue samples	Elevated in esophageal, colon, ovarian and lung tumors vs. normal tissues (48, 49). Expression was elevated with increasing stage of liver cancer and correlated with poor prognosis of gliomas and breast cancers (48).
	Serum	Higher in prostate cancer patients than healthy controls (48).
Transferrin receptor 2 (TfR2)	Cell lines	Upregulated in ovarian, colon, and glioblastoma cancer cell lines (50, 51)
	Tissue samples	Expression correlated with high tumor grade, but inversely correlated with prognosis of glioblastoma (51) and leukemia (52). Expressed in a proportion (~26%) of colon cancers (53).
Divalent metal transporter-1 (DMT1)	Tissue samples	Not detected in normal esophageal tissues, but overexpression of DMT1 was seen in tumors and associated with metastasis (49).
Clusters of differentiation 163 (CD163)	Tissue samples	>25% tumor cell positivity correlated with poorer survival of breast cancer patients (54).
Clusters of differentiation 91 (CD91)	Tissue samples	Highly expressed in breast, glioma, and endometrial tumors (55).
Ferritin (Ft)	Cell lines	Higher in more aggressive types of breast cancer cell lines (56).
	Tissue samples	FTH1 was overexpressed in esophageal adenocarcinoma (49). FTH1 and FTL highly expressed in HNSCC^*^ tissues compared to normal, associated with metastasis and high FTH1 resulted in shorter PFS (57). FTH1 and FTL higher in glioblastoma samples compared to normal brain, increased with glioma grade and correlated with worse survival (58). Higher FTL in metastatic lesions than primary melanomas (59). FTH1 and FTL were higher in ovarian tumor samples compared to benign and increased with tumor grade (60). High Ft associated with lymph node involvement and survival of breast cancers (61). Ft levels were elevated in colorectal cancers than normal colon mucosa (62).
	Serum	Higher in HNSCC patients with metastasis than without (57). Levels elevated compared to normal controls and associated with poor PFS for neuroblastoma (63), Hodgkinson's lymphoma (64), cervical (65), oral squamous cell (66), renal cell (67), T cell lymphoma (68), colorectal (62, 69), breast (70), and ovarian (60) cancers.
Ferroportin	Cell lines	Lower expression in prostate and breast cancer cells (71, 72).
	Tissue samples	Overexpressed in esophageal adenocarcinoma compared with normal (49). Expression was lower in prostate and breast cancers compared to normal and declined with increasing tumor grade (71–73). Low ferroportin expression levels in pancreatic cancer tissue were significantly associated with poor prognosis (74).
Hepcidin	Tissue samples	High expression observed in prostate and breast cancer tissues compared to normal (71–73).
Duodenal cytochrome b (Dcytb)	Tissue samples	Highly expressed in esophageal adenocarcinoma compared with normal (49). High Dcytb expression was associated with increased survival of breast cancer patients (75).
Iron regulatory protein-1 (IRP1)	Cell lines	Increased in some prostate and breast cancer cells (76, 77).
	Tissue samples	Decreased IRP1 expression hepatocellular carcinoma tissues compared to the adjacent non-tumorous liver tissues. Expression of IRP1 was significantly associated with disease stage and vascular invasion and low IRP1 associated with poor OS and PFS (78).
Iron regulatory protein-2 (IRP2)	Cell lines	Consistently increased in prostate and breast cancer cells (76, 77).
	Tissue samples	IRP2 expression is correlated with histologic grade and molecular subtype of human breast cancer (76). IRP2 was elevated in colorectal cancers compared to normal colon mucosa (79).

* *OS, Overall survival; PFS, progression-free survival; HNSCC, head and neck squamous cell carcinoma*.

### Iron Transport

The major iron transporters belong to the transferrin family including transferrin (Tf), melanotransferrin (MTf), and lactoferrin (Lf). Ovotransferrin is the avian equivalent and is a dietary source of Tf through consumption of eggs, but it is not endogenously expressed in humans. The carcinogenic role of transferrins depends on their saturation, whereby the apo (iron free) form can be chemopreventive/therapeutic by binding intracellular iron by reducing the LIP or holo (iron saturated) form it may be tumorigenic by acting as a source of iron for utilization by cancer cells. The following section describes the current knowledge for each of the human transferrins in the context of cancer.

Epidemiology studies measuring serum Tf established the link between high iron levels and cancer risk. As the major transporter of systemic iron, serum Tf is used as a marker of body iron levels. A study by Stevens et al. of >14,000 participants found that men with elevated saturated serum Tf (TS) were more likely to develop and die from cancer (80). Another cohort of >40,000 subjects observed levels exceeding 60% TS were highly correlated with colorectal (CRC) and lung cancer (35). Surprisingly, lower TS and higher iron-free Tf was observed in stomach cancers, which could be partly explained by *Helicobacter pylori* infection, which decreases iron absorption and iron is lost through hemorrhagic gastritis (81). Although most studies have measured serum Tf it is still unclear how well it correlates to levels of tumor Tf. Public data show that Tf mRNA is detectable in many cancers, but is highly enriched in liver cancer and although moderate cytoplasmic immunostaining for Tf protein was observed the vast majority was extracellular (www.proteinatlas.org). With liver being the main site of Tf synthesis it is not surprising that liver cancer tissue is enriched with Tf, but it remains to be determined whether liver cells remain the primary source of Tf for other cancers or whether tumor cells activate Tf synthesis independently to facilitate the transport of iron to the tumor microenvironment.

MTf was one of the first cell surface markers identified for melanoma. MTf can be membrane-bound or circulate in plasma (sMTf). Some liposarcomas, breast, and lung cancers also express MTf (37). MTf was highly expressed in CRC tissues, compared to normal adjacent tissue and in the serum of patients compared to healthy controls, suggesting potential as a diagnostic marker (38). Cell culture studies suggest that although MTf binds iron, it plays a minor role in cellular uptake (82). Characterization of MTf^−/−^ mice found no differences in the LIP compared to wild-type, nor changes in iron metabolism genes (83). However, engraftment of human melanoma cells with downregulated MTf had delayed tumor initiation and reduced growth in mice (83). MTf expression on melanoma cells also correlated with ability to transmigrate through brain endothelial cells to form brain metastases in mice (84, 85). This process is being explored to deliver therapeutic agents across the blood brain barrier (BBB) (86). The physiological relevance of sMTf is still unclear because of its inefficiency in donating iron compared to Tf and inability to bind transferrin receptors (87). However, sMTf has been found to promote cell migration and invasion through interaction with the urokinase-type plasminogen activator system *in vitro* and in a chick chorioallantoic membrane angiogenesis assay (85, 88). Taken together, MTf has both diagnostic and therapeutic implications and may play an important role in metastasis.

Lf is being investigated as a tumor suppressor through its role in iron sequestration. Lf has been implicated as both a tumor suppressor and potential chemotherapeutic, although whether the anti-cancer activity is related to its iron-binding capacity remains controversial (89, 90). Low Lf expression has been detected in gastric cancer (41) and nasopharyngeal (42) tumor tissues compared to normal. Hypermethylation of the Lf promoter has been observed in prostate cancer cell lines suggesting epigenetic silencing is a means of Lf loss in epithelial cells (39). Accordingly, Lf mRNA and protein expression was lower in prostate tumor cells, tissues, and serum of patients compared to normal (39). Although Lf is often not detectable in tumor tissues, Lf positivity correlates with good prognostic features including low Ki67 proliferation index and high progression-free and overall survival (40). Oral Lf (human and bovine) is being investigated as a chemopreventive and adjuvant therapy for several types of cancer. Lf supplement reduced growth, inhibited cell cycle progression and induced apoptosis of cancer cells *in vitro* (39, 91). Additionally, a clinical study of CRC patients receiving oral bovine Lf and chemotherapy had clinical benefit (92). Hence, Lf warrants further investigation as a prognostic marker and as a potential adjuvant cancer treatment.

Lipocalin 2 (LCN2), also known as neutrophil gelatinase-associated lipocalin (NGAL), is a secreted glycoprotein involved in iron trafficking. Increased LCN2 expression has been observed in ovarian (43), thyroid (44), breast (45, 93), lung (94), colon (46), and pancreatic (95, 96) cancers. In breast and thyroid cancers high LCN2 expression strongly correlated with advanced tumor grade and poor prognosis, but in ovarian, pancreatic and CRC it was associated well-differentiated tumors and a good prognosis (93). Overexpression of LCN2 in CRC cells suppressed proliferation, migration and invasion *in vitro* and tumor growth and metastasis *in vivo* (46). Similar tumor suppressive functions have been observed in liver cancer (47). Rather perplexing though, modulating LCN2 expression in human pancreatic cancer cells did not affect cell viability *in vitro*, but once engrafted LCN2-overexpressing tumors were smaller, poorly vascularized and had fewer metastases in an orthotopic nude mouse model (96). In contrast, in mice with diet-induced pancreatic cancer on a LCN2^−/−^ background had fewer and smaller tumors, less inflammation (reduced infiltration of CD45^+^ leukocyte cells and F4/80+ macrophages) and fibrosis compared to wild-type (95). Moreover, when murine tumor cells expressing LCN2 were implanted in LCN2 null mice, tumor growth was delayed and survival increased suggesting that expression in stromal cells within the tumor microenvironment is important for progression. As LCN2 null mice had lower expression of ferritin, and hence lower iron levels in pancreatic tissue, one explanation may be that the iron load of LCN2 determines its tumorigenic function. Rehwald et al. found that holo-LCN2 significantly induced migration and spheroid growth of renal cell carcinoma cells whereas iron-free LCN2 inhibited it (97). In sum, the role of LCN2 may be cancer-type specific and depend on iron saturation.

### Iron Uptake

Upregulation of TfR1 is often evident in cancers and promotes progression. As reviewed by Shen et al. TfR1 is overexpressed in leukemia, glioma, glioblastoma multiforme (GBM), breast, colon, liver, ovarian, prostate, and lung cancers, where it is correlated with poor clinical outcome and response to chemotherapy (48). Knockdown of TfR1 with a shRNA reduced proliferation and colony formation of pancreatic adenocarcinoma cells through impairing mitochondrial respiration and decreased ROS production (98). Likewise, antisense oligonucleotides against TfR1 inhibited tumor growth and lung metastases in the 4T1 mammary adenocarcinoma mouse model (99). Conflicting evidence is reported for CRC with histology showing TfR1 was elevated in tumors but associated with better survival rates and modifying TfR1 expression was said to promote growth, migration and invasion of CRC cell lines and suppress it in other reports (100–102). Most studies, indicate TfR1 is oncogenic, but there may be some circumstances where moderate TfR1 tumor expression is beneficial.

TfR2 is pro-tumorigenic by activating cell survival signaling rather than through importing iron. In contrast to the ubiquitously expressed TfR1, TfR2 is primarily expressed in liver and some cancer cells (50). TfR2 lacks an IRE sequence so its expression is not directly regulated by iron levels (103). TfR2 expression is primarily controlled through Tf where binding holo-Tf causes stabilization and recycling of the protein and apo-Tf induces lysosomal degradation. TfR2 binds holo-Tf with much lower affinity than TfR1 supporting its role as an iron-sensor rather than major importer (103). When TfR2 binds to holo-Tf, its internalization activates MAPK signaling which in turn mediates hepcidin synthesis (51). TfR2 is highly expressed in GBM and correlated with tumor grade, but inversely correlated with patient survival and TfR2 silencing in GBM cells inhibited proliferation and cell cycle progression (51). TfR2 was inversely correlated with leukemia tumor burden and overall survival (52). TfR2 is expressed in some colon cancer tissues and not normal colon epithelium, but was not associated with tumor grade (53). Thus, TfR2 could be involved in initiation and later adaptive mechanisms resulting in improved patient survival.

Three membrane iron transporters have been identified DMT1, Zrt-, and Irt-like protein 14 (ZIP14) and zinc transporter ZIP8 (ZIP8). DMT1 is important for iron uptake across the apical membrane of the gastrointestinal tract and intracellular endosomal membrane transport (6). Several reports suggest DMT1 is responsible for intracellular iron accumulation to support CRC proliferation (104–106). DMT1 was overexpressed in colon tumors compared to normal adjacent tissue and correlated with worse prognosis (104). Colon specific knockout of DMT1 reduced tumor burden in CRC mouse models (104, 105). DMT1 is also overexpressed in esophageal cancer (49). ZIP14 and ZIP8 are zinc transporters that also mediate cellular iron uptake through direct transport of NTBI across the cell membrane (5). Additionally, ZIP14 can export Tf-Fe from the endosome to the cytoplasm similar to DMT1. ZIP14 is important for uptake of NTBI especially by the liver and interestingly, knockdown of p53 which is known to alter iron metabolism, increased iron uptake by ZIP14 in HepG2 liver cancer cells (107). Research on ZIP8 has focused on its role in zinc transport in cancers or iron overload disorders and its iron-dependent role in cancer is unclear (108). For these transporters to internalize iron and, hence, drive iron-dependent cancer growth, iron must be in the reduced ferrous form.

The STEAP1-STEAP4 ferrireductases and cytochrome b reductase 1 (Dcytb) reduce iron for cellular uptake. Although STEAP1 does not possess metalloreductase activity, it co-localizes with the Tf-TfR1 complex in endosomes, suggesting that it still plays a role in iron metabolism. STEAP1 is overexpressed in several types of human cancer tissues and cell lines, including prostate, bladder, colon, pancreas, ovary, testis, breast, cervix, and Ewing sarcoma and has been implicated as a driver of cancer cell proliferation, invasion, and immune evasion (109). STEAP2 is overexpressed in prostate cancer tissues and knockdown of STEAP2 in cell lines inhibits proliferation, cell cycle progression and induces apoptosis through regulation of the MAPK pathway (109). STEAP3 regulates vesicular trafficking and its interaction with several targets can inhibit cell cycle progression and induce apoptosis (109). Likewise, STEAP4 is also involved in trafficking, where its expression is induced by several cytokines and, thus, plays a role in inflammation, however, information regarding its role in cancer is vague (109). Dcytb reduction of ferric iron is important for absorption by duodenal enterocytes, but it has also been identified as a predictor of outcome and chemotherapy response for breast cancer patients (75). In conclusion, ferrireductases play an important role in iron uptake and in doing so contribute to cancer progression.

Heme-bound iron is taken up by scavenger receptors clusters of differentiation 163 and 91 (CD163 and CD91). Although CD163 is primarily expressed on monocytes and macrophages it has been detected on tumor cells with high malignant potential. However, tumor cell lines do not express CD163, even after stimulation with macrophage activating cytokines, so it has been hypothesized that tumor cells fuse with macrophages becoming more genetically unstable and aggressive (110). Assessment of patient tissues with CD163-positive tumor cells correlated with higher tumor grade, invasiveness, radioresistance and poor progression free- and overall survival in melanoma (111), breast (54, 112, 113), CRC (113), renal cell (114), and gastric (115) carcinomas. CD91 is overexpressed in breast, gliomas, and endometrial carcinomas and low expression of CD91 was correlated with low metastatic potential of liver cancers (55). Knockdown studies in gliomas established it as a modulator of cancer cell proliferation, migration, invasion and apoptosis through regulation of MAPK, Akt, c-Jun N-terminal kinases (JNK), and nuclear factor κ B (NF-κB) oncogenic signaling (55). Therefore, heme scavengers contribute to tumorigenesis.

Serum ferritin is a diagnostic and prognostic cancer biomarker for some cancers. Ferritin is often elevated in the serum of cancer patients including those with neuroblastoma (63), Hodgkinson's lymphoma (64), cervical (65), oral squamous cell (66), renal cell (67), T cell lymphoma (68), CRC (69), and breast (70) cancers and were often associated with increased tumor grade and shorter survival. Tumor-associated macrophages (TAMs) are proposed to be the major source of high serum ferritin in cancer patients. TAMs synthesize and secrete ferritin into the microenvironment to metabolically reprogram the cancer cells stimulating proliferation, angiogenesis and immunosuppression in a paracrine manner (116). TfR1 has been considered as an importer of FTH1 in humans, while scavenger receptor class A member 5 (SCARA5) was identified as the importer for FTL. SCARA5 is downregulated in cancers and correlated with high tumor grade, metastasis and poor survival (117). Stable overexpression of SCARA5 inhibited proliferation, migration and invasion and promoted cell cycle arrest and apoptosis in breast cancer cells (117). Although iron poor, TAM-derived serum ferritin stimulates proliferation of cancer cells in an iron-independent manner which may account for anti-intuitive correlation of SCARA5 and cancer suppressive affects.

### Iron Storage

Ferritin sub-unit expression and intracellular distribution determine prognosis. For instance, immunostained GBM tissues had elevated FTL and mRNA expression correlated with poor survival, whereas no statistical difference was evident for FTH1 (55). Additionally, immunofluorescence of GBM cell lines showed FTL was mainly localized in the nucleus. In melanoma samples, high FTL was detected in primary and metastatic lesions by immunohistochemistry but was exclusively cytoplasmic (59). Both FTL and FTH1 stained strongly in head and neck cancer tissues compared to normal and higher expression was observed with metastasis, however further analyses of public data found FTL had no prognostic significance but high FTH1 mRNA predicted poor survival (57). Ovarian cancer samples had elevated FTL and FTH1 detected in the cytoplasm and nucleus compared to benign tissue and increased with tumor grade (60). Interestingly, in triple negative breast cancer samples high cytoplasmic and total FTH1 was correlated with favorable prognosis, whereas high nuclear expression was a poor prognostic factor (118). In breast cancer cell lines expression of FTH1 and FTL mRNA and protein were low in cells with an epithelial phenotype and high in cells with a more aggressive mesenchymal phenotype (56). Immunofluorescence and immunoblotting of subcellular protein fractions confirmed the accumulation of chromatin-bound nuclear FTH1 in mesenchymal MDA-MB-231 cells and a decrease in intracellular iron (56). It was suggested nuclear FTH1 was protective of the DNA from free iron-induced toxicity and promoted a more aggressive phenotype.

The multi-functional role of ferritin becomes increasingly evident by modifying its expression. In two separate studies downregulation of FTL with an antisense construct (59) and FTH1 with shRNA (119) in melanoma cells inhibited proliferation and invasion *in vitro* and tumor growth *in vivo* (59). In melanoma cells FTL is necessary to resist oxidative stress-induced apoptosis (59). Similarly, mesothelioma cells overexpress FTH1 to protect against asbestos-induced ROS and its knockdown rendered them more sensitive to apoptosis (120). In contrast, transient overexpression of FTH1 in non-small cell lung carcinoma cells triggered apoptosis (121). A cancer stem cell model of GBM showed downregulation of both subunits inhibited growth of gliomaspheres and prevented tumor formation in mice (58). FTH1 has been linked to drug resistance in breast (56) and ovarian (122) cancer wherein downregulation of FTH1 increased chemosensitivity. Additionally, FTL and FTH1 bind the anti-angiogenic molecule high molecular weight kininogen (HKa), preventing its dimerization, necessary for its functional activity and consequently, promoting endothelial cell survival, migration, adhesion, and angiogenesis to support tumor growth (123, 124). In summary, ferritin subunit expression and localization determine its role in cancer biology and must be tightly controlled.

When levels exceed ferritin storage capacity, iron may be stored in the form of hemosiderin, a complex of ferritin aggregates, denatured proteins and lipids. Hemosiderin deposits are most commonly observed in macrophages, particularly in the liver and spleen, sites that are important for maintaining iron homeostasis and following hemorrhage, suggesting that its formation may be related to the breakdown of red blood cells and hemoglobin (125). These deposits can be stained by Prussian blue iron (III) and visualized by light microscopy (126). When placed in an external magnetic field hemosiderin, like ferritin displays superparamagnetism. This is particularly useful for imaging using MRI as the iron deposits cause high T2^*^ contrast. Deposits of TAMs-laden with hemosiderin can be mapped using MRI and quantification of the frequency and size can be used as a non-invasive marker of disease stage, imaging of metastases and to assess the success of iron-based therapy (125, 126).

### Iron Utilization

Iron is utilized in the synthesis of Fe-S and heme and as an enzyme co-factor. Because Fe-S proteins act as a source of iron, there is a complex system that ensures Fe-S clusters are assembled correctly, trafficked to specific apoproteins, and remain protected during these processes. Drugs that interfere with Fe-S metabolism and destabilize the cluster can be effective at inhibiting the growth of cancers (8). Such an example is β-phenethyl isothiocyanate, an inhibitor of leukemia cell growth, in part by producing ROS which degrade the Fe-S center of NADH dehydrogenase 3 from respiratory complex I and subsequently suppresses mitochondrial respiration (8). Cyclooxygenase-2 (COX-2) is a heme-containing enzyme that is usually undetected in healthy tissues, but its expression is induced during inflammation and is highly expressed in some cancers and accordingly, COX-2 selective inhibitors have elucidated its role in cell growth and survival, angiogenesis, cell invasion and inflammation (127). These are merely two examples of proteins that utilize iron, but many more exist whose disarray can contribute to cancer and represent potential therapeutic targets.

### Iron Export

Being the sole exporter of intracellular iron, intuitively reduced ferroportin in cancer cells promotes iron accumulation. Ferroportin expression is lower in prostate and breast cancer cells compared to normal epithelial cells (71, 72). Prostate cancer tissues had decreased ferroportin reactivity with increasing histological grade (71). Ferroportin was markedly reduced in breast cancer tissue compared to normal and associated with reduced progression-free patient survival (72). Furthermore, overexpression of ferroportin reduced human and mouse mammary tumor growth and metastasis (73, 99). *In vitro*, 4T1 mouse mammary cells with inducible ferroportin expression had reduced colony forming ability, underwent cell cycle arrest and apoptosis (73). In pancreatic cancer samples high hepcidin expression and lower ferroportin staining were associated significantly shorter overall survival (74). As reduced ferroportin is seemingly a consequence of increased hepcidin-induced receptor degradation, the hepcidin-ferroportin axis represents an attractive target to inhibit tumor growth and metastasis.

### Iron Regulatory Proteins

Cellular iron homeostasis is predominantly controlled by the IRE-IRP system, accordingly, altered expression of IRPs is associated with cancer. IRP1 and IRP2 have distinct phenotypes. For example, both IRP1 and IRP2 are overexpressed in breast cancer, but only knockdown of IRP2 decreased the LIP and inhibited mammary tumor growth in mice (76). In human lung cancer cells with inducible IRP1 overexpression there was no impact on proliferation *in vitro*, but when implanted *in vivo* had suppressed tumor growth (128). In hepatocellular carcinoma (HCC), IRP1 expression was lower in tumor than normal adjacent tissues, correlated the tumor stage and predicted overall and recurrence-free survival (78). In prostate cancer cells IRP2 was consistently overexpressed and knockdown inhibited growth *in vitro* and *in vivo*, while IRP1 was detected in some cell lines and knockdown only modestly reduced proliferation *in vitro* (77). IRP2 was overexpressed in colon cancer tissues compared to normal and interestingly, correlated with BRAF mutations and it was confirmed *in vitro* that IRP2 overexpression was driven by hyperactivation of the MAPK pathway (79). Therefore, the expression and roles of IRPs in cancer differ by tumor type.

## The Role Of Iron in Metastasis

Several Fenton reaction-based carcinogenesis and metastasis models have elucidated the role that iron plays in cancer progression. Generation of ROS through Fenton reactions can promote cancer stem cell-aggressiveness, cell migration, and angiogenesis. Welch and Hurst recently described that in addition to the hallmarks of cancer in general, such as genetic instability, sustained proliferation, resistance to programmed cell death, evasion of immune killing etc., there are also several features that are required for cancer dissemination (3). These have been termed the hallmarks of metastasis and include motility and invasion, ability to modulate the secondary site or local microenvironments, plasticity, and ability to colonize secondary tissues (3). Iron has been linked to the hallmarks of metastasis and the following sections highlight the potential of exploiting iron metabolism to treat or prevent metastasis.

### Evolution of Tumor Cells Toward a Metastatic Phenotype

For tumors to grow the cells must survive and proliferate despite the fail-safe mechanisms that should prevent them. Cancer cells must be highly adaptable to somewhat hostile microenvironments (hypoxic, low nutrient availability, acidic extracellular space, etc.). These harsh conditions coupled with genetic instability give rise to tumor heterogeneity. Tumor heterogeneity is illustrated by studies of single-cell clones originating from a single tumor which display diverse phenotypes including differences in metastatic potential (3). Iron-induced oxidative stress modifies the genome, epigenome, and proteome, giving rise to tumor heterogeneity and evolving metastatic potential. In a rodent model, rats were repeatedly administered ferric nitrilotriacetate which induced widespread genomic alterations that led to the development of tumors (129). Iron treatment of colonocytes caused widespread hypomethylation, especially to oncogenes belonging to Akt, MAPK, and EGFR pathways and subsequently their expression increased (130). Iron overload as a result of hemochromatosis, causes aberrant hypermethylation of genes characteristic of HCC, suggesting iron-induced epigenetic modification could be an early event in malignant transformation (131). Treatment of breast cancer cells with the iron chelator desferrioxamine (DFO) caused significant global epigenetic alterations and decreased expression of several histone demethylases (132). Although DNA methylation is aberrant during different stages of disease progression specific iron-induced pro-metastatic changes remain to be clearly defined.

### Motility and Invasion

Cell migration is the movement of cells to a different position within tissue in response to attractive or repulsive stimuli. Cells can become motile without directionality by autocrine secretion of motility factors or are directed by following a gradient of factors via chemotaxis. The types of cell movement (mesenchymal, amoeboid, or collective) are influenced by extracellular cues but can switch between modes to adapt to the local microenvironment. Increases in the intracellular LIP stimulate migration and invasion of cancer cells. Kim et al. found that the activity of heme-oxygenase 1 (HO-1), a known metastasis promoter, increased intracellular iron levels inducing migration and invasion of breast cancer cells (133). Similarly, prostate cancer cells with higher iron levels as a consequence of hepcidin-induced ferroportin degradation had higher migratory capacity compared with knockdown of hepcidin (134). Furthermore, iron loading of lung cancer cells with ferrous sulfate (FeSO_4_) induced migration and invasion *in vitro* (135). Iron contributes through activating pro-migratory signaling pathways, remodeling the ECM and produces an environment conducive to metastatic spread.

Cell movement can be associated with morphological changes known as epithelial-mesenchymal transition (EMT), whereby cells lose their polarity and cell-cell adhesions and reorganize their cytoskeleton and shape. EMT arises from changes in gene expression with downregulation of epithelial markers (e-cadherin, occluding, and claudins) and upregulation of mesenchymal markers including transcription factors (Snail, Slug, ZEB, Twist) and factors required for motility and invasion (N-cadherin, vimentin, and fibronectin) (3). EMT can be induced by iron-loading or reversed by iron deprivation. For example, ferric chloride treatment caused colon cancer cells to transition to a mesenchymal phenotype, with decreased e-cadherin-mediated cell-cell junctions and increased invasiveness, whereas treatment with the iron chelator DFO restored e-cadherin localization, cells then became more compact and epithelial-like and had significantly reduced invasion (136). Treatment of esophageal cancer cells with the iron chelator deferasirox (DFX) inhibited migration and invasion of cells in transwell chemotaxis assays as well as reduced invasion of 3D tumor spheroids through suppressing N-cadherin (137). In contrast, Chanvorachote and Luanpitpong found that although iron induced a lung cancer stem cell phenotype this was not associated with EMT (138). Treatment of cancer cell lines with FeSO_4_ had little change in morphology and expression of epithelial and mesenchymal proteins even after 7 days extended treatment. Therefore, EMT was either not critical in iron-induced lung cancer stemness and/or it may depend on the model used.

Migration and invasion of cancer cells are controlled by signaling pathways. Cooperation of transforming growth factor β (TGF-β) and Wnt/β-catenin signaling pathways promotes EMT and is regulated by iron. Canonical Wnt signaling is mediated by β-catenin, which functions in the formation of the adherens junctions by complexing with e-cadherin linking it to the actin filaments of the cytoskeleton. β-catenin is usually maintained at low levels, but upon activation by Wnt ligands, it accumulates and translocates into the nucleus and cooperates with transcription factors including the TGF-β-induced SMAD complex and increases expression of mesenchymal factors (139). Several studies have shown that iron supplementation increases expression of TGF-β and its receptors triggering SMAD transcriptional activity, as well as stabilizing β-catenin supporting its accumulation and, hence, activating target gene expression (139–141). Alternatively, iron depletion reverses cells to an epithelial-like state, thereby reducing EMT-related migration, invasion and metastasis (142–146). Interestingly, Chen et al. reported the iron chelator Dp44mT suppressed colon cancer cell viability, migration, invasion, and reversed TGF-β-induced EMT through activation of Wnt/β-catenin as they observed increased target gene expression (147). In contrast, Li et al. showed that Dp44mT reduced migration and metastasis *in vivo*, but reduced β-catenin, c-myc, and cyclin D1 (148). Thus, crosstalk between TGF-β and Wnt/β-catenin signaling is well-established to promote EMT-induced migration and invasion, but downstream activity may depend on context.

Iron remodels the ECM to enable motility and invasion of cancer cells. Matrix metalloproteinases (MMPs) are the key proteolytic enzymes involved in the degradation of the ECM. LCN2 forms a complex with MMP-9 and its overexpression promotes EMT of breast cancer cells with enhanced migration and invasion *in vitro* and when implanted in mammary fat pads had increased local invasion and metastasis to lymph nodes (149). Ferric ammonium citrate (FAC) supplementation of head and neck cancer cells increased expression of MMP-9, a known promoter of tumor invasion, through activation of MAPK and Akt pathways (150). Although FAC passively passes through the cell membrane independently from the transferrin uptake pathway and further, the impact on migration and invasion was not investigated (150). Iron-induced hydroxyl radicals and lipid peroxides increased expression of MMP-1 and MMP-3 after UVB irradiation of dermal fibroblasts, which cleave/degrade interstitial collagens, proteoglycans and structural glycoproteins, while DFO treatment degraded these MMPs (151). Likewise, Dp44mT abrogates the induction of gelatinase activity (MMP-2) and inhibited liver tumor invasion (144). Therefore, several lines of evidence show that excess iron induces expression of MMPs which are required for degradation of the ECM and iron chelation may be a promising strategy to prevent invasion of cancer cells.

### Modifying the Local Tumor Microenvironment

Behavior of resident and recruited cells within the tumor microenvironment is influenced by iron levels. In addition to remodeling the ECM, iron modulates the local tumor environment by recruiting new cells, suppressing the immune response, and altering the behavior of cells to complete the metastatic cascade. For tumors to progress they must grow, recruit or coopt blood vessels to provide oxygen and nutrients. Under hypoxic conditions, culture of breast cancer cells with low-iron containing media promoted proangiogenic signaling via vascular endothelial growth factor (VEGF) and treatment of capillary endothelial cells with the conditioned media stimulated their elongation and extension toward a vascular morphology (152). Treatment of endothelial cells with FAC inhibited autocrine VEGF signaling, cell proliferation, migration, tube formation and sprouting in culture and systemic administration repressed tumor angiogenesis *in vivo* (153). In contrast, treatment of rat brain endothelial cells with LCN2 increased intracellular labile iron, generating ROS and stimulated scratch migration and tube formation in Matrigel (154). Oxidative stress is a well-established promoter of angiogenesis (155). Therefore, too much or too little iron can induce migration of endothelial cells and encourage neo-vascularization.

Tumor infiltrating inflammatory cells are educated by tumor cells to adopt an iron-donor phenotype which promotes tumor growth and spread. For example, secretion of GM-CSF by tumor cells stimulates secretion of Tf by neutrophils and drives tumor growth and metastasis (156). In the early stages of tumorigenesis pro-inflammatory cytokines prompt M_1_-like macrophages to sequester iron and produce ROS as an anti-tumor defense mechanism, but chronic inflammation and immune tolerance can lead to M_2_-like TAMs releasing iron to support tumor progression (157). Interestingly, iron chelation can shift TAMs from the iron-donor to iron sequestration phenotype (158). M_2_-like macrophages express high levels of ferroportin, however, its knockdown in cultured macrophages did not inhibit the release of iron into the supernatant, nor did it inhibit the proliferation-stimulating effect of the supernatants on breast cancer cells (159). In fact, it was demonstrated that tumor cells undergoing apoptosis release sphingosine 1-phosphate (S1P) which stimulates the secretion of holo-LCN2 by macrophages (159, 160). Holo-LCN2 accelerated proliferation and migration of lymphatic endothelial cells in 2D culture and promoted lymph vessel sprouting in 3D models (160). In animal studies, macrophage-derived LCN2 stimulated lymphangiogenesis and promoted metastasis of breast tumors (160). Interestingly, high intracellular iron and FTH1 suppressed cell surface expression of major histocompatibility complex (MHC) class 1 on tumor cells and macrophages and consequently, iron depletion rendered tumor cells highly susceptible to death by natural killer (NK) cells (161). In accordance, decreasing iron in the tumor microenvironment increased the destruction of breast cancer cells by NK cells (162). Therefore, recruited cells acquire an iron-release phenotype to support the growth, migration, neo-vascularization and immune evasion.

Iron has immunosuppressive effects on the adaptive immune system. For instance, patients with hemochromatosis have altered CD4^+^ and CD8^+^ T cell proportions and decreased cytotoxic T lymphocytes, rendering them susceptible to infections, impaired cancer immune surveillance and autoimmune conditions (163). Iron also directly modulates T cell surface molecules including CD4 and CD2 (164). Excess intracellular iron initially stimulates the proliferation of T and B lymphocytes, but ultimately leads to cell death from oxidative stress (165). T cells rapidly expand during an immune response and their proliferation is dependent on iron availability. Therefore, upregulation of TfR1 on the surface of T cells is a very early event in immune activation (157). Furthermore, conditional knockout studies of FTH1 in bone marrow of mice revealed impaired maturation of B and T subsets and found that proliferation of these cells was dependent on intracellular storage of iron in ferritin (165). Interestingly, FTH1 released by tumor cells has immunosuppressive effects of lymphocytes. It appears that FTH1 mediates its effects by upregulating the expression of CD86 and B7-H1 on dendritic cells, which in turn interact with CTLA4 and PD-1 on T cells, respectively, and induce the secretion of interleukin 10 (IL-10) and interferon γ (IFN-γ) to inhibit antigen-specific immune responses against tumors (166). Tumor cell-derived LCN2 cooperates with C-C Motif Chemokine Ligand 2 (CCL2) to suppress immune cells by decreasing the costimulatory molecule HLA-DR and increasing expression of immunosuppressive programmed death ligand 1 (PD-L1) on CD11c^+^ regulatory dendritic cells, which is followed by induction of CD4^+^/FOXP3^+^ regulatory T cells and tumor escape (167). Hence, iron is essential for immune cell survival but in excess leads to defects in tumor recognition by immune cells and immune tolerance.

Cancer-associated fibroblasts (CAFs) represent the majority of non-cancer cells within the stroma of solid tumors and are derived from resident fibroblasts or through differentiation of other precursor cells. CAFs acquire a constitutively activated state (myofibroblast-like), whereby metabolic and phenotypic changes allow CAFs to supply nutrients and metabolites, creating a fertile microenvironment to support tumor growth and metastasis. ROS are key regulators in TGF-β-mediated fibroblast-to-myofibroblast transition which release cytokines, growth factors and ECM remodeling factors and increases tumor invasiveness (168). Targeting Wnt/β-catenin can also impair TGF-β-induced myofibroblast transition, again linking these two signaling pathways and their role in promoting metastasis through modulating the tumor microenvironment (169). High levels of hepcidin are often observed in tumors to maintain an iron-utilization phenotype within tumor cells. CAFs can induce hepcidin in tumor cells through interleukin 6 (IL-6) secretion and stimulation of signal transducer and activator of transcription 3 (STAT3) signaling (170), illustrating one of the mechanisms where iron metabolism underpins tumor-stroma crosstalk.

### Metabolic Plasticity

Metabolic plasticity allows cancer cells to survive and metastasize through the ability to switch between different forms of energy production depending on substrate availability. Recently it has been shown that cells maintain the ability to switch between metabolic phenotypes very rapidly and use both OXPHOS and glycolysis mechanisms to overcome hostile environments in the body and even develop resistance to drugs (171). For example the initial accumulation of ROS drives cancer cells to switch from OXPHOS to glycolysis, but chronic ROS exposure rewires metabolism toward the pentose phosphate pathway with cells undergoing a stem-like phenotype that's more resistant to therapies (172). Lactate and pyruvate, the byproducts of glycolysis, regulate hypoxia-inducible factor 1α (HIF-1α) and Wnt signaling independently of oxygen availability, which in turn alters iron metabolism (34). Hypoxia activates HIF-1α and enhances tumor iron accumulation by upregulating expression of TfR1 and HO-1 which degrades heme to release iron, and ceruloplasmin which oxidizes iron to facilitate Tf iron loading (34). Iron deficiency also mimics hypoxia, causing stabilization of HIF-1α and promoting EMT, cell migration and invasion. Interestingly, in normoxic conditions DFO-induced iron deficiency was reported to promote EMT in colon cancer cells, and increase migration and invasion in contrast to other reports (173). Although, recently it was found that DFO induces mitochondrial iron accumulation which generates ROS and, therefore, enhanced migration/invasion was driven by mitochondrial ROS (100). Consistent with this finding, only particular types of ROS induce cancer cell migration, where FeSO_4_-generated ·OH promoted lung cancer cell migration, but treatment with ${\text{O}}_{2}^{-}$ or H_2_O_2_ inhibited it (135). Therefore, metabolic switching may be responsible for altering tumor iron metabolism and promoting metastasis.

### Colonization of Secondary Sites

Before cells break away from the primary tumor mass, they communicate with the environment in distant organs to establish a pre-metastatic niche. This phenomenon was recognized because particular types of tumors favor dissemination to certain organs, but not others and this is directed partly through factors secreted from the primary tumor (174). These soluble factors are sometimes found in extracellular vesicles called exosomes (175). Exosomes also serve as carriers of other cellular material including DNA, lipids, proteins, mRNAs, and non-coding RNAs (176). Exosomes isolated from metastatic rat adenocarcinoma BSp73ASML cells contained ferritin light and heavy polypeptide mRNA and these exosomes modulated the pre-metastatic niche to support colonization of the poorly metastatic cells in lymph nodes and lungs of rats (177). Although, this study relied on animal models and didn't validate ferritin protein expression as a carrier of iron in the exosomes. However, in further support of this finding, ferritin (FTH1 and FTL) has been detected in exosomes isolated from bladder, ovarian, nasopharyngeal, and prostate and hepatocellular carcinomas in humans (www.exocarta.org). Therefore, ferritin contained within exosomes could act as a carrier of iron to create a favorable “soil” for cancer cells to “seed.”

Once tumor cells disseminate, they may start to proliferate and form a secondary mass or lie dormant for months or even years. Dormant tumor cells are largely refractory to targeted or conventional therapies and to date, our knowledge of the biology underlying tumor dormancy is limited. Temporary cell cycle arrest, coordination of quiescence and autophagy, a dormancy-permissive microenvironment, immunosuppression and epigenetic factors have been linked to tumor cell dormancy (178). Poor nutrient and oxygen availability within the microenvironment cause cancer cells to secrete factors that inhibit the Akt pathway, resulting in slowly proliferating, quiescent cells and induction of pro-survival autophagy (178). Interestingly, iron chelating agents such ciclopirox olamine (CPX) and VLX600, have been shown to inhibit growth of both proliferating and quiescent cancer cells (179). Through chelation of iron, activity of the iron-dependent enzymes that form part of the electron transport chain become impaired resulting in mitochondrial dysfunction (180). In order to meet energy demands, HIF-1α becomes stabilized and activates glycolysis. In some cells pro-survival autophagy is induced to uptake glucose and other nutrients to fuel energy production. However, for parts of the tumor with poor vascularization, and hence, poor access to extracellular nutrients bioenergetic demands cannot be met triggering cell death (180). It is the lack of metabolic plasticity of tumor cells within poorly vascularized regions of the tumor microenvironment that allows iron chelating agents to inhibit quiescent cells and present attractive therapeutic opportunities for metastatic disease, especially in combination with other chemotherapeutic agents.

## Iron Modulation as a Cancer Therapy

Several iron modulators, which were initially developed for other conditions, are being repurposed to treat cancers. Iron chelators are summarized in Table 2 along with their mechanism of action and clinical testing status in the context of cancer.

**Table 2:** Iron chelating agents under clinical development for treatment of cancers.

**Compound**	**Mechanism**	**Cancer(s)**	**Development status**	**Clinical results**
Bp44mT	Thiosemicarbazone (BpT series), synthetic iron chelator	Neuroblastoma, lung	Preclinical	N/A
Ciclopirox olamine (CPX)	Hydroxypyridinone, synthetic iron chelator	Hematological, advanced solid tumors	Phase I	(181)
Curcumin	Polyphenol, plant-derived iron chelator	Various	Phase I–III	(182)
Deferasirox (ICL670A, DFX)	Tridentate triazole, synthetic iron chelator	Hepatocellular carcinoma, hematological	Phase I–II	(183)
Deferiprone (DFP)	Hydroxypyridinone, synthetic iron chelator	Prostate	Pre-clinical	N/A
Desferrioxamine (DFO)	Siderophore, natural iron chelator	Neuroblastoma, leukemia, hepatocellular carcinoma	Phase I	(184)
Dp44mT	Thiosemicarbazone (DpT series), synthetic iron chelator	Various	Pre-clinical	N/A
DpC	Thiosemicarbazone (DpT series), synthetic iron chelator	Advanced solid tumors	Phase I	Not yet published
Epigallocatechin gallate (EGCG)	Catechin gallate, natural iron chelator	Colon, prostate	Phase I–II	(185)
Silybin	Flavonolignan, natural iron chelator	Prostate, lung, hepatocellular carcinoma	Phase I–II	(186–188)
Tachpyridine	Hexadentate, synthetic iron chelator	Various	Pre-clinical	N/A
Triapine	Thiosemicarbazone, synthetic iron chelator	Various	Phase I–III	(189–191)
VLX600	Triazinoindolyl-hydrazone, synthetic iron chelator	Advanced solid tumors	Phase I	(192)

### Iron Chelation

Therapeutic iron chelating agents were initially developed to treat iron overload. For many years DFO was the standard iron overload treatment and was later found to have anti-cancer activity. Studies of DFO using leukemia and neuroblastoma cell cultures showed promising results eventually leading to clinical testing of patients with these cancers. Although most patients showed partial or complete responses, its short half-life and poor solubility required patients to undergo long periods of subcutaneous infusion, with frequent pain and swelling at the site of injection and oral alternatives were pursued. Deferasirox (Exjade, Jadenu, DFX) is an oral iron chelator implemented for iron overload and is currently being trialed for hematological malignancies. DFX was effective against leukemia cells in preclinical studies and because leukemia patients receive repeated blood transfusions, DFX offers a dual benefit as an anti-cancer agent and treatment for iron overload complications. Additionally, DFO and DFX are effective in preclinical studies of pancreatic (193), breast (194), liver (183), gastric (195), and esophageal (196) cancers, and because they are well-characterized they are often used as positive controls for the study of other iron modulators. Despite promising preclinical results, a pilot study of DFX in advanced hepatocellular carcinoma patients found dose-limiting toxicities and the majority (4/5) of the patient tumors progressed while on treatment, therefore the efficacy of DFX for the treatment of solid tumors remains questionable (183).

Deferiprone (DFP) is an oral metal chelator approved for the treatment of β-thalassemia and has been investigated in preclinical cancer studies. Investigation into the pharmacotoxicity profile revealed that in addition to chelating iron, thereby reducing the LIP, the compound also had redox activity which resulted in the production of intracellular ROS (197). In breast cancer cell lines, it was demonstrated that due to its small flat aromatic structure, DFP gains access to and chelates the Fe^2+^ ion at the active sites of iron-dependent histone lysine demethylases (198). These enzymes control gene transcription by modifying the epigenome, silencing tumor suppressors and activating transcription of oncogenes and promote the growth of cancers. DFP was effective at reducing prostate cancer growth in Myc-CaP and TRAMP-C2 orthotopic mouse models, although the efficacy was dependent on initial tumor iron levels which accumulated from infiltrating hemosiderin-laden macrophages, thus highlighting the dependency on iron for DFP activity (125).

Ciclopirox olamine (CPX) is a fungicide that has additional anti-microbial, anti-inflammatory and anti-cancer activity. Its anti-tumor activity is mediated through iron chelation and subsequent inhibition of iron-dependent enzymes such ribonucleotide reductase, reduced signaling through the EGFR/P-Akt, DOHH/eIF5A, and Wnt/β-catenin pathways, and modulating cell cycle regulators (199). Recently, CPX was shown to downregulate DJ-1, an oncogene that functions as an endogenous antioxidant, resulting in the accumulation of ROS, impairing mitochondrial function and inducing apoptosis of CRC cells (200). CPX inhibited growth of several cancer cell lines (including rhabdomyosarcoma, head and neck, lung, breast, and CRC) more overtly than normal non-transformed cells, such as primary dermal fibroblasts, peripheral blood mononuclear cells, lymphocytes, and mucosal epithelial cells (200–203). Oral CPX treatment inhibited growth of leukemia (203), breast (204), neuroblastoma (202), pancreatic (201), and CRC (200) tumors in the mice. Continual administration of CPX with a subcutaneously implanted pump prevented metastasis of neuroblastoma tumors in mice (202). CPX was evaluated in a phase I trial of hematological malignancies and was well-tolerated with some clinical effect seen in two thirds of patients (181). However, efforts to further progress CPX were abandoned because of the poor solubility of the drug, its rapid metabolism into an inactive glucuronide and quick clearance from the body. A phosphoryloxymethyl ester-based prodrug of ciclopirox (CPX-POM) has since been developed which has improved hydrophilicity and protects the site of glucuronidation to improve bioavailability. CPX-POM has demonstrated efficacy in preclinical models of bladder cancer (205) and a phase I trial (NCT03348514) has been initiated for patients with advanced solid tumors.

Tachpyridine is a synthetic metal chelator that binds iron, zinc, and copper. Tachpyridine inhibits the growth of multiple cancer cell types, induces apoptosis and selectively sensitizes cancer cells to ionizing radiation (206, 207). A screen of 55 cancer cell lines from the National Cancer Institute (NCI) panel had a mean GI50 of 5.7 μM (207). Tachpyridine induced cell cycle arrest in G2 phase in HeLa and CRC cells, whilst an analog missing iron binding ability could not, suggesting therapeutic activity was iron-dependent (206). This was further supported by a study of bladder cancer cells treated with tachypyridine derivatives that were able to bind zinc and copper, but not iron and lost cytotoxicity compared to the parental compound (208). Further investigation revealed long term exposure of cultured bladder cancer cells depletes iron but also induces oxidative stress through redox cycling of the tachpyridine–iron complex (209). Tachpyridine induced apoptosis of breast cancer cells, but pre-treatment with iron or zinc abrogated this effect (210). To date, tachpyridine is yet to be tested for efficacy in cancer models *in vivo*.

Thiosemicarbazones were among the first metal chelators to be evaluated specifically for their anti-cancer potential. Screening of various thiosemicarbazone derivatives has prioritized compounds that are resistant to glucuronidation and rapid elimination, the most successful thus far is triapine. Several studies have attributed its mechanism-of-action to potent inhibition of RNR and, hence, reduced DNA replication and repair (211). It has broad-spectrum anti-cancer activity having been tested against in mouse models of leukemia, lung and ovarian cancers (211). Interestingly, triapine crosses the blood-brain barrier and effectively killed leukemia cells implanted in the cerebellums of mice (211), indicating it would be an effective treatment for disseminated disease. To date, triapine has undergone 28 phase I and II clinical cancer trials and is currently in a phase III trial (NCT02466971) for cervical cancer in combination with cisplatin and radiotherapy with expected completion 2023. Published results report high doses of the drug caused dose-limiting toxicities, so combination therapies that involve administration of lower individual drug doses is preferable. Results of a phase II study in female reproductive cancers found addition of triapine to the cisplatin and radiotherapy regimen resulted in a 92% complete response rate, compared to 69% without, and increased the 3-years progression free survival from 77 to 92% (212). Importantly, elevated methemoglobin levels in red blood cells, a concern seen in other trials with high dose triapine, was not observed in this cohort, suggesting a tolerable and effective dose has been identified which could pave the way for treatment of other cancers (212).

Further analog development using thiosemicarbazones as a structural basis has resulted in various series of compounds with several outstanding in terms of iron-binding affinity and anti-proliferative activity. The NT series was a group of compounds based on the parental ligand 2-hydroxy-1-naphthylaldehyde thiosemicarbazone (NT). These were screened in neuroepithelioma cells with three standout compounds NT, N4mT, N44mT, which showed anti-proliferative activity in additional cancer cell lines, but to a lesser extent in normal cells, such as fibroblasts or macrophages (213). Based on the success of triapine the DpT series was developed, of which Dp44mT showed high iron chelation and anti-proliferative activity. However, its toxicity profile has somewhat mired its clinical progression. Dp44mt caused cardiotoxicity and weight loss in mice so efforts to progress to the clinic were halted (214). Following the DpT series was the evaluation of aromatic substituents, namely 2-benzoylpyridine thiosemicarbazone (BpT) series, which demonstrated enhanced growth inhibition and redox-cycling activity (215). Bp44mT was also effective for inhibiting growth of lung cancer xenografts with no noticeable cardiotoxicity (216). To date though DpC, an analog of Dp44mt is the most potent and well-tolerated compound showing efficacy both *in vitro* and *in vivo* in models of pancreatic (217), neuroblastoma (218), and lung (214) cancers. DpC has undergone phase I clinical testing for advanced solid tumors, the results of which will be greatly anticipated.

A screen for drugs that preferentially target quiescent cells in colon cancer spheroids identified VLX600 as an ideal candidate. Although the precise mechanism of action was unknown at the time, analysis using the Connectivity Map database determined iron chelators CPX and DFO produced similar gene expression profiles, suggesting iron chelation was the mode of action for VLX600 (179). Indeed, compound modeling and subsequent cell culture studies with/without iron supplementation confirmed the cytotoxicity of VLX600 was attributable to iron chelation. In contrast to other iron chelators, such as the thiosemicarbazones, VLX600 does not induce ROS (180). Instead, through inhibition of iron-dependent complexes (I, II and IV) of the electron transport chain VLX600 impairs mitochondrial OXPHOS limiting the metabolic plasticity of tumor cells (180). VLX600 showed efficacy in *in vitro* and *in vivo* models of CRC at very low concentrations (0.5–16 mg/kg) with minimal toxicity observed (180). A phase I study was initiated to evaluate VLX600 in patients with advanced solid tumors, however due to slow recruitment the trial was terminated early. In total 19 patients were enrolled, all received at least one dose of VLX600 and the drug was well-tolerated at all doses (192). The study was underpowered so no efficacy endpoints were met, and the maximum tolerated dose and recommended phase II dose could not be determined. Thus, although initial safety and tolerability profiles suggest VLX600 warrants further clinical investigation it remains to be seen whether this is pursued.

Remarkably, some natural compounds with anti-cancer properties were found to act through iron chelation. For instance, epigallocatechin-3-gallate (EGCG) extract, the major green tea polyphenol has potent anti-proliferative effects in colon cancer cells attributed to its antioxidant and free iron scavenging activity (219). However, ingestion of green tea or ECGC extract does not produce clinically relevant cytotoxic levels of EGCG in plasma, so nanodelivery systems are being explored as a means of increasing stability and bioavailability (220). Silybin, derived from the milk thistle plant (*Silybum marianum*), acts as an antioxidant through iron chelation and shows additional anti-inflammatory activity through suppression of NF-κB, induces apoptosis and cell cycle arrest and inhibits angiogenesis and metastasis (221). Curcumin, derived from the plant *Curcuma longa*, has long been used in traditional medicine, but has also shown efficacy against colon, duodenal, stomach, esophageal, and oral cancers (222). In fact, several clinical trials have been initiated or are ongoing evaluating safety and efficacy of curcumin as an adjuvant therapy for various cancers (www.clinicaltrials.gov). However, it is only in the last decade that the therapeutic effects of curcumin were discovered to be related to iron chelation (222). The therapeutic potential of natural compounds as chemotherapeutics and for chemoprevention have been recognized and it is interesting that iron chelation has been identified as a major mechanism of action.

### Targeting Iron Metabolism and Regulatory Mechanisms

Elevated TfR1 and its internalization mechanism, positions the receptor as a desirable therapeutic target and drug-delivery strategy. A number of strategies have been developed for targeting the TfR1 including its natural ligand Tf, targeting peptides, monocolonal antibodies, and antibody fragments (scFv) (223). These may directly antagonize the receptor (e.g., anti-TfR1 antibodies) to induce cytotoxicity or a non-neutralizing method can be utilized for receptor-mediated internalization of drugs. Many clinical trials have been conducted with anti-TfR1 antibodies and show some evidence of anti-tumor efficacy, but immunogenicity remains a major concern (224). scFv fragments and peptides interact specifically with the extracellular domain independently of Tf-Fe binding and their small size offers better solid tumor penetration (225, 226). TfR1 targeting molecules may be directly conjugated to therapeutic cargo or nanoparticles encapsulating therapeutic agents. For instance, transferrin conjugated to doxorubicin had enhanced cytotoxicity in drug-resistant leukemia cells compared to free drug, but did not accumulate normal human fibroblasts indicating improved tumor specificity (227). MBP-426 is a liposomal carrier conjugated to Tf in a phase II clinical trial as a delivery agent for oxaliplatin to treat gastric and esophageal adenocarcinomas (NCT00964080). A phase II trial is evaluating SGT-53, a cationic liposome with TfR1-scFv encapsulating a wild type p53 sequence that will be used in combination with gemcitabine and paclitaxel (NCT02340117). Similarly, SGT-94 uses the same targeted system to deliver a modified form of the retinoblastoma tumor suppressor gene, RB94, and has recently completed phase I assessment (NCT01517464). Another fascinating drug delivery mechanism involves a pro-drug strategy via trioxolane conjugation that reacts with ferrous iron in the tumor microenvironment to activate drug release (228). It is hoped that these delivery strategies will circumvent systemic toxicity and preliminary results seem promising.

miRNA expression is often altered in cancers and miRNA replacement or antagonization represent potential therapeutic strategies. TfR1 expression is highly elevated in HCC and shows an inverse correlation with miR-148a and miR-152 expression and their ectopic overexpression suppressed growth of HCC cells (229, 230). miR-7-5p is a potent tumor suppressor of HCC growth including in models of sorafenib resistance (231). miR-7-5p expression was reduced in pancreatic adenocarcinoma samples and loss of miR-7-5p was proposed to permit TfR1-driven cell proliferation and metabolism (232). Interestingly, miR-7-5p and miR-141-3p were found to target IREs within 3′-UTR of TfR1, thereby reducing its mRNA and protein expression by competing with the IRE-IRP system (232). Although, this finding was later disputed by another research group and requires further clarification (233). One explanation could be that alternative splicing gives rise to isoforms lacking IRE sequences that are subject to different control mechanisms. For example, DMT1 encodes four splice variant transcripts including one lacking an IRE sequence (DMT1B-nonIRE) of which, let-7d was confirmed to specifically target and consequently reduced its expression in erythroleukemia cells (234). Elevated nuclear FTH1 in breast cancer cells, as a result of reduced miR-200b was proposed to protect DNA against oxidative damage, therefore, miR-200b replacement sensitized the cells to the DNA-damaging agent doxorubicin (56). Ferroportin expression is reduced in lung cancer patients, was negatively correlated with miR-20a level and was confirmed as a target using cell lines *in vitro* (235). miR-485-3p expression is elevated during iron deprivation and it targets ferroportin to reduce iron export, suggesting an antagomiR could suppress iron accumulation (236). As our knowledge of miRNAs that regulate iron homeostasis expands more therapeutic targets may emerge and will be further realized by the clinical development of RNA-based therapeutics.

### Ferroptosis

Ferroptosis was recently identified in 2012, as an iron-dependent form of regulated cell death with characteristics different to other forms of cell death. Activation of ferroptosis is dependent on the intersection of amino acid, lipid and iron metabolism (2). The defining features of ferroptosis are the presence of oxidizable phospholipids acylated with polyunsaturated fatty acids (PUFA-PLs), redox-active iron and defective or inhibited lipid peroxide repair mechanisms (2). Ferroptosis was identified from screens that detected small molecule inhibitors which were lethal to cultured tumor cells, but the mechanisms were distinct from known programmed death pathways (237). Later was discovered that CD8^+^ T cells activate tumor ferroptosis during treatment with anti-CTLA4 and anti-PD-L1 immunotherapies (238). Furthermore, both immunotherapy and radiotherapy independently initiate ferroptosis, yet when combined act synergistically sensitizing tumors and improving tumor control (239). The mechanism was attributed to the release of IFNγ from CD8^+^ T cells which impaired tumor cell uptake of cysteine by system Xc^−^ and resulted in iron-dependent lipid peroxidation and ferroptosis.

Ferroptosis inducers (FINs) are classified into four classes, class I which inhibit system Xc^−^, class II which directly inhibit glutathione peroxidase 4 (GPX4), class III indirectly inhibit GPX4, and class IV increases iron levels. GPX4 is an enzyme that reduces lipid peroxides, however when GPX4 activity is impaired, free iron catalyzes lipid peroxides to form toxic lipid ROS. For a detailed description of class I-III FINS refer to Yang et al. (240), Dixon and Stockwell (2), and Lu et al. (237). Although, it is classed as type II, withaferin A, a natural ferroptosis-inducing agent, increases the intracellular iron pool and inhibited growth of neuroblastoma xenografts (241). Likewise, artesunate, an anti-malarial, interacts with lysosomal iron and generates ROS leading to ferroptosis. Phase I trials of artesunate in various malignancies showed improved recurrence-free survival (242, 243) and repurposing efforts continue to progress in the clinical pipeline. The only known class IV FIN is ferroptosis inducer endoperoxide (FINO_2_) which causes ferrous iron oxidation, however, the half-maximal effective concentration (EC_50_) for a renal cancer cell line and two immortalized fibroblast cell lines was 20 μM and it has not been investigated *in vivo* so its utility may be limited.

Iron levels determine the sensitivity of cells to ferroptosis. The expression of iron regulatory genes (e.g., TfR1, Tf, ferritin, and ferroportin) determine the sensitivity of cells to ferroptosis and this is positively correlated with intracellular iron levels (244, 245). Lysosomes are iron rich and accordingly, treatment of breast cancer cells with the lysosome disruptor siramesine increased intracellular iron and ROS, thereby triggering ferroptosis (245). Furthermore, extracellular iron (from high-iron diets or iron treatments) sensitize cells to ferroptosis (246). In serendipitous fashion iron-based nanoparticles which were developed for other purposes also show anti-cancer potential. For instance, iron saturated ferritin nanoparticles loaded with doxorubicin induced ferroptotic death in cultures of leukemia, CRC, breast, liver, cervical, and lung cancer cells which overexpress TfR1 (247). Furthermore, iron-based nanoparticles which are already approved to treat iron deficiency, are used for imaging tumors and in preclinical studies as drug delivery carriers also show therapeutic benefit. Ferumoxytol, a commercially available formulation of iron oxide nanoparticles, show anti-cancer activity against mammary tumors and prevent lung and liver metastases in mouse models (248). Interestingly, the iron acts as a chemoattractant for macrophages which release hydrogen peroxides into the tumor microenvironment which react with the iron and inhibit growth and spread of the tumor (248). Therefore, iron nanoparticles present a major opportunity for cancer therapy and diagnosis.

### Combination Therapies

Understanding the biology that underlies therapeutic resistance has identified opportunities for iron modulators to exploit these mechanisms and enhance tumor responses. For example, one of the known causes of multidrug resistance is efflux of chemotherapeutics from the cancer cells by upregulating expression of drug-transporters, such as P-glycoprotein (Pgp). Pgp-mediated drug resistance occurs by its rapid internalization, redistribution and increased expression through HIF-1α activity; this facilitates accumulation of the drug in lysosomes, creating a “drug safe house” away from its therapeutic target, and then eventual efflux from the cancer cell (249). Dp44mT and DpC overcome resistance to doxorubicin and vinblastine (250, 251) by utilizing lysosomal Pgp transport, where the compounds complex with lysosomal iron, generate ROS which disrupt the lysosomal membrane and induces apoptosis (252, 253). When Dp44mT was combined with paclitaxel, 5-fluorouracil, doxorubicin, tamoxifen, and 4-hydroperoxycyclophosphamide *in vitro* the drugs synergistically enhanced cytotoxicity of breast cancer cells (254). Given the positive proof-of-concept results with Dp44mT, if the results of the DpC clinical trial are encouraging the next logical step would be to assess it in combination with existing cancer treatments.

Other iron chelators are being evaluated in combination with a common chemotherapeutic cisplatin Triapine is being assessed in several clinical trials in combination with cisplatin (www.clinicaltrials.gov). Results published to date indicate that the drug combinations are safe and may improve progression-free survival (212). Triapine was reported to enhance the response to cisplatin by disrupting homologous recombination repair following cisplatin-induced DNA damage (255). Given this unique mechanism sequential combination of triapine and cisplatin therapy is necessary to achieve synergism, indicating the schedule for administering the drugs is important for efficacy (255). However, some controversy exists around the mechanism of cisplatin and its role in iron metabolism. Guo et al. suggested that cisplatin induces ferroptosis through depleting glutathione (GSH) and inactivating glutathione peroxidases (256). Their rationale was that treatment with the ferroptosis inhibitor ferrostatin-1, DFO, or IRP2 knockdown, partially reversed cisplatin-induced toxicity and visually, mitochondrial changes were observed consistent with ferroptosis. But the results are not overly convincing with very mild changes evident when cisplatin-induced toxicity was “reversed.” Another study reports that cisplatin depletes cancer cells of iron by directly binding to IRP2, inhibiting its binding to IREs and as a result increased ferritin and decreased TfR1 expression, thus lowering the LIP (257). Additionally, combination of cisplatin and DFO enhanced cytotoxicity through augmented iron depletion both in cell culture and xenografts of colon cancer cells in mice (257). Given the strength of evidence reported it seems likely cisplatin reduces intracellular iron rather than triggering iron-dependent cell death, but further research should provide some clarity.

In contrast to the plethora of studies investigating iron chelators with chemotherapies, there is limited evidence for whether they increase the efficacy of targeted and immune-based therapies. A reason proposed for why iron chelators haven't been successful for some solid tumors is because of HIF-1α stabilization and increased expression leading to increased proliferation, angiogenesis and metastasis. Therefore, a strategy to improve efficacy of iron chelation is dual treatment with a specific HIF-1α inhibitor. This was the approach taken by Lang et al. where they combined DFO and lificiguat (also named YC1) and observed synergistic reduction in cell viability of pancreatic cancer cell lines (258). They then used a liposome-based delivery system cross-linked with transferrin to codeliver DFO and YC1, targeting pancreatic tumors with expression of TfR1. The nanoparticles improved the circulation half-life compared to free DFO, facilitated uptake of the drugs by tumor cells and once released DFO and YC1 exerted a synergistic anti-tumor effect in both subcutaneous and orthotopic pancreatic cancer xenografts. This study highlights the power of combination therapy and using targeted delivery systems to improve bioavailability and biological activity. Despite the recent success of immunotherapy, particularly with respect to PD-1/PD-L1 checkpoint inhibitors, evidence for combined iron chelators and immunotherapies is lacking. Given the role of iron metabolism in the tumor microenvironment this area should emerge as a hot topic for further investigation.

Several FINs increase chemosensitivity. For example, erastin increases sensitivity to chemotherapies (e.g., temozolomide, cisplatin, cytarabine/ara-C, and doxorubicin/Adriamycin) in certain cancer cells (237). PRIMA-1, a non-genotoxic agent that targets mutant/deleted p53 and activates ferroptotic cell death, enhanced anti-tumor activity of dexamethasone and doxorubicin in multiple myeloma xenografts (259). GSH activity is important for detoxification of chemotherapeutics, and hence the GSH specific inhibitor buthionine sulfoximine (BSO) has subsequently been evaluated in clinical trials in combination with the chemotherapeutic melphalan in advanced malignancies (252, 253). BSO and combined therapy was well-tolerated and showed some biological activity, although it is not clear whether any clinical responses are due to ferroptosis induction or inhibiting drug detoxification (252, 253). These agents show proof-of-principle that ferroptosis determines chemosensitivity represent an attractive for new cancer drug discovery.

## Concluding Remarks and Future Directions

The field of tumor iron metabolism is complex and dynamic with new discoveries being made about how it is regulated, its involvement in cancer progression, advances in the development of iron-disruption therapeutics and methods of exploiting it for imaging and drug delivery. For many years cancer had been considered as a genetic disease. However, further investigation has revealed that cancers display abnormal metabolism with many features of a metabolic disorder. Altered iron metabolism is a feature observed in tumors which increase iron influx and reduce efflux from tumor cells to support their survival, rapid cell division and metastasis. However, it is still unclear whether dysregulated iron metabolism precedes transformation or is a consequence of it, acting as an adaptive mechanism for tumor progression. Unless iron-induced oncogenesis can be prevented, the order is irrelevant, and the treatment approach would be the same.

The dual role of iron in cancer has highlighted the potential of iron modulation as a strategy to treat advanced cancers, but the question remains whether it is best to inhibit iron utilization or to flood cells with iron and induce ferroptosis. Seemingly, there are limitations and toxicity concerns for each approach which may need to be overcome for iron modulation to be an efficacious treatment. There also remains controversy in the literature about the complications of iron supplementation during cancer treatments. Cancer patients often become anemic whilst undergoing chemotherapy, but there is the potential that iron supplementation could increase tumorigenicity and promote drug resistance. There is a fine line in ensuring iron homeostasis that will need to be taken into consideration in the management of cancer patient care. Particularly, because there is no “magic bullet” for treating metastatic disease and therapeutic resistance is common. Ultimately, patient care will require a multi-pronged approach and therefore identifying and optimizing novel combinatorial strategies and taking iron levels into account will improve outcomes for patients with advanced cancer.

Although there have been some major advances in the development of iron-based therapeutics their toxicity, short-half life, rapid metabolism, and emerging resistance are ongoing concerns. A lack of insight into the mechanisms underlying resistance to these therapies has somewhat hampered generation and optimization of new analogs to overcome these issues. Metabolic studies will likely provide the information we need for determining the route and sites of drug “de-activation” and whether pro-drug strategies could circumvent it. It may also inform novel drug combinations to improve tumor responses or help identify which patients are most likely to benefit from iron-based therapies. Furthermore, targeting strategies, such as bioconjugation or use of nanoparticle systems to deliver iron-modulators may be developed to improve bioavailability, tumor specificity and could be especially useful for crossing the BBB to treat metastatic disease. The future, thus, looks bright for the more widespread introduction of iron-based therapies into mainstream oncology, but most likely in a precision medicine personalized care basis.

## Author Contributions

RB provided the concept, wrote most of the manuscript, and illustrated the figures. KR and TK wrote and edited the manuscript. RG and DT discussed and edited the manuscript. PL provided the concept, discussed, and edited the manuscript.

### Conflict of Interest

The authors declare that the research was conducted in the absence of any commercial or financial relationships that could be construed as a potential conflict of interest.

## Abbreviations

BBB: blood brain barrier
BSO: buthionine sulfoximine
CAFs: cancer-associated fibroblasts
CCL2: C-C Motif Chemokine Ligand 2
CD163: clusters of differentiation 163
CD91: clusters of differentiation 91
c-Myc: proto-oncogene c-Myc
COX-2: cyclooxygenase-2
CPX: ciclopirox olamine
CRC: colorectal cancer
Dcytb: cytochrome b reductase 1
DFO: desferrioxamine
DFP: deferiprone
DFX: deferasirox
DMT1: divalent metal ion transporter 1
DOHH: deoxyhypusine hydroxylase
ECM: extracellular matrix
EGCG: epigallocatechin-3-gallate
EGFR: epidermal growth factor receptor
eIF5A: eukaryotic initiation factor 5A
EMT: epithelial-mesenchymal transition
EV: extracellular vesicles
FAC: ferric ammonium citrate
FINs: ferroptosis inducers
FINO2: ferroptosis inducer endoperoxide
FTH1: ferritin heavy chain 1
FTL: ferritin light chain
GBM: glioblastoma multiforme
GSH: glutathione
GPX4: glutathione peroxidase 4
HCC: hepatocellular carcinoma
HIF-1α: hypoxia-inducible factor 1α
HKa: high molecular weight kininogen
HO-1: heme-oxygenase 1
IFN-γ: interferon γ
IL-6: interleukin 6
IL-10: interleukin 10
IRE: iron-responsive element
IRP1: iron regulatory protein 1
IRP2: iron regulatory protein 2
JNK: c-Jun N-terminal kinases
LCN2: lipocalin 2
Lf: lactoferrin
LIP: labile iron pool
MAPK: mitogen-activated protein kinases
MHC: major histocompatibility complex
miRNA: microRNA
MMPs: matrix metalloproteinases
MtFt: mitochondrial ferritin
MTf: melanotransferrin
mTOR: mammalian target of rapamycin
NF-κB: nuclear factor-κB
NGAL: neutrophil gelatinase-associated lipocalin
NK: natural killer
NRF2: nuclear factor erythroid 2-related factor 2
NTBI: termed non-transferrin bound iron
PCBP2: poly(C)-binding protein 2
PD-L1: programmed death ligand 1
Pgp: P-glycoprotein
PUFA-PLs: polyunsaturated fatty acids
RNR: ribonucleotide reductase
ROS: reactive oxygen species
S1P: sphingosine 1-phosphate
SCARA5: scavenger receptor class A member 5
STAT3: signal transducer and activator of transcription 3
STEAP3: six-transmembrane epithelial antigen of the prostate 3
TAMs: tumor-associated macrophages
Tf: transferrin
TfR1: transferrin receptor 1
TfR2: transferrin receptor 2
TGF-β: transforming growth factor β
UTR: untranslated region
VEGF: vascular endothelial growth factor
ZIP14: Zrt- and Irt-like protein 14
ZIP8: zinc transporter ZIP8.
